# Influenza and tuberculosis co‐infection: A systematic review

**DOI:** 10.1111/irv.12670

**Published:** 2019-09-30

**Authors:** Sibongile Walaza, Cheryl Cohen, Stefano Tempia, Jocelyn Moyes, Athermon Nguweneza, Shabir A. Madhi, Meredith McMorrow, Adam L. Cohen

**Affiliations:** ^1^ Centre for Respiratory Diseases and Meningitis National Institute for Communicable Diseases of the National Health Laboratory Service Johannesburg South Africa; ^2^ School of Public Health, Faculty of Health Sciences University of the Witwatersrand Johannesburg South Africa; ^3^ Influenza Division Centers for Disease Control and Prevention Atlanta GA USA; ^4^ Influenza Program Centers for Disease Control and Prevention Pretoria South Africa; ^5^ Medical Research Council, Respiratory and Meningeal Pathogens Research Unit University of the Witwatersrand Johannesburg South Africa; ^6^ Department of Science and Technology/National Research Foundation: Vaccine Preventable Diseases University of the Witwatersrand Johannesburg South Africa; ^7^ U.S. Public Health Service Rockville MD USA; ^8^ Global Immunization Monitoring and Surveillance, Expanded Programme on Immunization Department of Immunization Vaccines and Biologicals World Health Organization Geneva Switzerland

**Keywords:** influenza, interaction, tuberculosis

## Abstract

**Introduction:**

There are limited data on risk of severe disease or outcomes in patients with influenza and pulmonary tuberculosis (PTB) co‐infection compared to those with single infection.

**Methods:**

We conducted a systematic review of published literature on the interaction of influenza viruses and PTB. Studies were eligible for inclusion if they presented data on prevalence, disease association, presentation or severity of laboratory‐confirmed influenza among clinically diagnosed or laboratory‐confirmed PTB cases. We searched eight databases from inception until December 2018. Summary characteristics of each study were extracted, and a narrative summary was presented. Cohort or case‐control studies were assessed for potential bias using the Newcastle‐Ottawa scale.

**Results:**

We assessed 5154 abstracts, reviewed 146 manuscripts and included 19 studies fulfilling selection criteria (13 human and six animal). Of seven studies reporting on the possible effect of the underlying PTB disease in patients with influenza, three of four analytical studies reported no association with disease severity of influenza infection in those with PTB, whilst one study reported PTB as a risk factor for influenza‐associated hospitalization.

An association between influenza infection and PTB disease was found in three of five analytical studies; whereas the two other studies reported a high frequency of PTB disease progression and complications among patients with seasonal influenza co‐infection.

**Conclusion:**

Human analytical studies of an association between co‐infection and severe influenza‐ or PTB‐associated disease or increased prevalence of influenza co‐infection in individuals' hospitalized for PTB were not conclusive. Data are limited from large, high‐quality, analytical epidemiological studies with laboratory‐confirmed endpoints.

## 1 INTRODUCTION

Influenza virus infections cause substantial annual morbidity and mortality in humans worldwide.^1^, ^2^, ^3^ Globally, it is estimated that annual influenza epidemics result in three to five million cases of severe illness and between 290 000 and 650 000 influenza‐associated respiratory deaths.^4^, ^5^ In 2015, there were an estimated 10.4 million incident cases of tuberculosis and 1.8 million tuberculosis deaths globally.^6^ In 2015, tuberculosis was the most common cause of infectious disease‐related deaths worldwide, with the majority of cases reported in Asia and Africa.^6^

Both influenza and tuberculosis impair host immune responses. Specifically, influenza can impair T‐cell immunity and weaken innate immune responses against secondary bacterial infections.^7^, ^8^, ^9^, ^10^, ^11^, ^12^ Lethal synergism associated with viral and bacterial infections can result in increased risk of influenza‐associated mortality.^13^ Furthermore, individuals with pulmonary tuberculosis (PTB) may be at increased risk for severe influenza disease and death due to chronic lung disease and immunosupression. Ecological studies and mathematical modelling of epidemiologic data suggest an increase in the frequency of influenza disease or severe influenza‐associated disease in individuals with PTB during influenza pandemics^14^, ^15^, ^16^, ^17^, ^18^ or during seasonal influenza epidemics^19^ compared with otherwise healthy individuals.

Influenza infection may facilitate the progression of latent *Mycobacterium tuberculosis* infection to tuberculosis disease and alter the clinical presentation of tuberculosis.^20^ It is also possible that influenza infection may exacerbate PTB.

Whilst chronic lung diseases are a known risk factor for severe outcomes due to influenza infection and influenza vaccination is recommended in this group, PTB is not listed as a separate priority group.^21^ Understanding the interaction between influenza and PTB may assist in determining whether individuals with PTB should be prioritized for influenza vaccination and treatment with antiviral medications. We conducted a systematic review of published literature on the association between laboratory‐confirmed influenza and PTB, that is influenza in individuals with tuberculosis and tuberculosis in individuals with influenza infection, in order to summarize whether co‐infection affects presentation, progression or disease outcome.

## 2 METHODS

We conducted a systematic review, which is reported in accordance with PRISMA guidelines,^22^ to summarize whether individuals with co‐infection present with severe influenza or PTB disease as compared to those with single infection or disease. Burden, transmission and severity of co‐infection were included for completeness.

### 2.1 Eligibility and inclusion criteria

**Figure 1:**
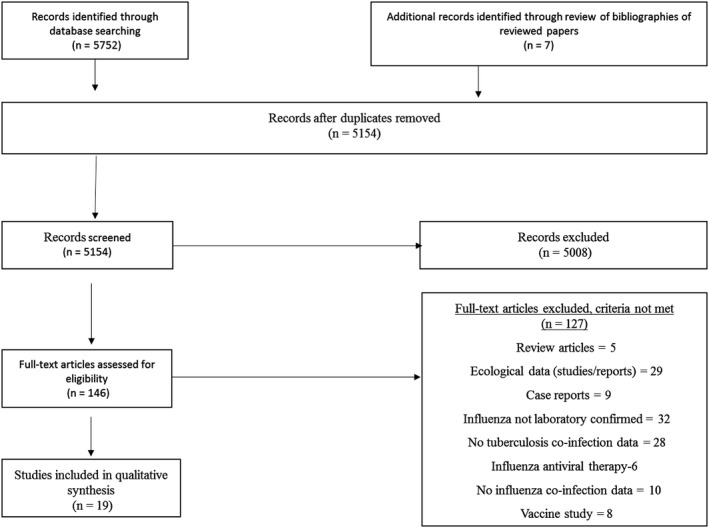
Flow diagram for systematic review of influenza and tuberculosis co‐infection

This review was restricted to published abstracts and articles from inception to December 2018 that reported data on the association (burden of disease, transmission and severity) between laboratory‐confirmed influenza and clinically diagnosed or laboratory‐confirmed PTB. Due to the scarcity of published data, descriptive studies, including studies without comparison groups, were included. Articles that included seasonal or pandemic influenza and animal experimental studies were also included. For human studies, inclusion was limited to studies in which influenza was laboratory‐confirmed and tuberculosis included PTB. Animal studies were included as they may provide useful insights into possible underlying mechanisms of interactions in humans. Studies that modelled ecological data on the association between influenza and tuberculosis, individual case reports, vaccine studies and influenza antiviral therapy in patients with tuberculosis were not included. Study selection is summarized in Figure 1.

### 2.2 Search strategy

We conducted a systematic review of the scientific literature identified through searches using online databases. For our search, we included terms for influenza (“influenza” or “flu” or “influenza virus” or “human influenza”) and for tuberculosis (“tuberculosis” or “TB”). The Medline, Embase, PsycINFO, CINAHL, Web of Science, Cochrane, CAB Abstracts and Global Health databases were searched. The search strategy, which was completed in consultation with a research librarian, differed slightly by database (Appendix S1). In addition, bibliographies of papers that were reviewed were checked for further relevant publications. The search was restricted to articles published in English, French, Italian, German, Russian, Finish, Japanese or Portuguese.

### 2.3 Study selection

Literature search results (titles and abstracts) were screened independently by two authors (SW and one of the co‐authors: CC, AN, JM, ALC or MM) to identify all citations that possibly met the inclusion criteria. Full manuscripts of selected citations were retrieved and assessed by one reviewer (SW) against the inclusion/exclusion criteria and checked independently by a second reviewer, one of the co‐authors (ALC, MM, JM, AN, CC, ST). Additional articles were identified from reviewing bibliographies of published articles. Discrepancies in included articles were resolved by consensus between the two reviewers with involvement of a third reviewer (CC) where necessary. Animal experimental studies, descriptive and analytic studies in humans were included. Studies that reported data on the association between laboratory‐confirmed influenza and clinically diagnosed or laboratory‐confirmed PTB including the following were included:

- Prevalence and risk for influenza‐associated severe disease among patients with PTB disease;
- Prevalence and risk for PTB‐associated severe disease among patients with influenza infection;
- Effect of influenza on PTB disease progression
- Clinical presentation of influenza and PTB co‐infection; and
- Immune response to co‐infection, presentation or outcome of influenza‐tuberculosis co‐infection in animal studies.

### 2.4 Data extraction and synthesis

Data extracted from each study were entered into a Microsoft Excel worksheet, including: year published, study design, type of study (descriptive vs analytical), location of study, period of study, sample size, study setting (hospital/ICU/outpatient), type of influenza testing, tuberculosis testing method (microscopy, culture, polymerase chain reaction [PCR]), results (influenza and tuberculosis), influenza strains, outcome and findings. In studies where only a number of cases or percentage was reported, we calculated the counterpart for the review. We summarized data under two groups, PTB in patients with influenza and influenza in patients with PTB; this was decided after examination of data. We did not stratify by age, gender or other characteristics.

Individual studies were independently assessed for potential bias or confounding. When studies used either cohort or case‐control designs, we used the Newcastle‐Ottawa Scale to rate the quality of the included papers.^23^ Studies were considered high quality if the Newcastle‐Ottawa Scale was ≥7 out of 9 and were considered of low quality if the score was ≤3 out of 9. Study methods differed; summary measures (odds ratios, relative risks), when reported, were abstracted. Data synthesis consisted of reporting the key findings of the different studies. Where possible the studies were classified according to whether they fall among the 22 high tuberculosis burden countries (HBC) that account for aproximately. 80% of world's tuberculosis cases.^3^ Review protocol attached (Appendix S2).

### 2.5 Ethics

Since this study used published data, it was exempt from human subjects ethics review.

## 3 RESULTS

The search identified 5752 records; 598 of these were duplicates and were removed. Seven additional records were identified through other sources (Figure 1). The remaining 5154 titles and abstracts were screened. Of these, 146 articles were identified for full review, and 19 articles met the inclusion criteria. Of these, 13 were in humans and six were animal experimental studies.

### 3.1 Human studies

Of the 13 human studies, 10 used real‐time reverse transcription polymerase chain reaction (RT‐PCR) and three used unpaired serology to test for influenza infection. A total of 27 566 individuals (range 19^24^‐12 196^25^) were included in human studies, 12 777 (range 31^26^ to 12 196^25^) in descriptive and 14 789 (range 19^24^ to 3646^27^) in analytical studies (Tables 1 and 2). Eight of the human studies were analytical.^24^, ^27^, ^28^, ^29^, ^30^, ^31^, ^32^, ^33^ Eight studies had laboratory‐confirmed results for both influenza and tuberculosis (Tables 1 and 2). Nine studies were from high burden countries including six from Africa. In addition, three studies from Europe reported data from a period (1952‐1963) with high tuberculosis prevalence. Three of the eight analytical studies were of high quality. Six studies reported on PTB disease in individuals with influenza,^24^, ^25^, ^27^, ^29^, ^34^, ^35^ six on influenza in individuals with the underlying PTB^26^, ^28^, ^31^, ^32^, ^33^, ^36^ and one on both (Tables 1 and 2).^30^

**Table 1:** Summary of studies reporting on effect of tuberculosis in individuals with influenza

Study	Country	Year	Descriptive/ analytical	Objective/hypothesis	Setting	Number studied	Influenza type	Laboratory‐confirmed influenza	TB diagnosis	Findings	Association^a^	Newcastle‐Ottawa Score^b^
Archer, (2009)^34^	South Africa	2009	Descriptive	Describe epidemiology of lab‐confirmed pandemic influenza cases in South Africa	Hospital admissions	72 cases	Pandemic A(H1N1)2009	RT‐PCR	Lab methods not specified as diagnosed/ reported by attending clinician	7/72 (10%) cases who died with influenza A(H1N1)pdm09 had TB vs 1% TB prevalence in 2006 in general community. Not evaluated statistically	NA	NA
Puvanaligam (2011)^35^	India	2009‐ 2010	Descriptive	Describe clinical profile of H1N1 cases	Hospital admissions	442 cases	Pandemic A(H1N1)2009	RT‐PCR/culture	Not specified	8.8% influenza A (H1N1)pdm09 cases had TB vs 0.4% TB prevalence in general population (*P* < .001)	NA	NA
Abadom^27^	South Africa	2009‐2012	Analytical	Assessed risk factors for influenza‐associated SARI hospitalization	Hospital admission	3646	Seasonal and pandemic	RT‐PCR	Microscopy, *M tuberculosis* culture/XPert MTB/RIF	TB was a risk factor for influenza‐associated hospitalization (CPR 1.85, 95%CI 1.68‐2.02) Covariates adjusted for: history of smoking (CPR 3.8, 95% CI 3.5‐4.16); HIV (CPR 3.61, 95% CI 3.5‐3.71; admission in past 12 mo (CPR 2.07, 95% CI 1.92‐2.23), age, 3rd dose of pneumococcal vaccine in <5 y (CPR 0.74, 95% CI 0.741‐0.7.0).	Yes	6
Noh (2013)^25^	South Korea	2009‐ 2011	Descriptive	Describe cases with concurrent TB and influenza	Hospital admissions	12 196 subjects	Pandemic A(H1N1) 2009	RT‐PCR	Auramine stain, TB PCR and TB culture	No deaths in the 7 cases of concurrent influenza‐TB infection. Not evaluated statistically	NA	NA
Ope (2011)^29^	Kenya	2007‐2009	Analytical	Describe risk factors for influenza hospitalization	Hospital admissions	64 cases; 190 controls	Seasonal (AH3N2 &A H1N1 & B)	RT‐PCR	Self‐report verified by clinician diagnosis and medication	TB associated with influenza hospitalization on bivariate (OR 12.0, 95% CI 1.3‐107.37) but not on multivariate (aOR not presented) analysis Adjusted for chronic lung disease, chronic heart disease, HIV infection, owns cattle and number of chickens owned. HIV‐infected more likely to be hospitalized for influenza [aOR3.56 (95% CI 1.25‐10.07)]	No	8
Roth (2013)^30^	Thailand	2003‐2011	Analytical	Compare characteristics of TB/influenza to influenza and TB only	Hospital admissions	7180 subjects	Seasonal & pandemic (AH1N1) 2009	RT‐PCR	AFB ± culture	Influenza‐TB co‐infection not associated with increased severity/mortality. Deaths in 0/23 co‐infected vs 17/604 (2.8%) with influenza only (*P* = 1.0) HIV prevalence among co‐infected 3/18 (17%)	No	6
Koegelenberg, (2009)^24^	South Africa	2009	Analytical	Describe epidemiological characteristics, clinical features and outcome of pandemic H1N1 cases complicated by respiratory failure	Intensive care unit admissions	19 cases	Pandemic A(H1N1) 2009	RT‐PCR	Lab methods not specified‐presence/absence of disease	4/19 ICU cases with influenza A(H1N1)pdm09 had TB. TB in 4/13 (31%) who died vs 0/6 who survived, (*P* = .5)	No	6

Abbreviations: AFB, acid‐fast bacilli; AOR, adjusted odds ratio; CPR: case‐population ratio; lab, laboratory; NA, not applicable; OR, odds ratio; RT‐PCR, real‐time reverse transcriptase polymerase chain reaction; SARI, severe acute respiratory illness; TB, tuberculosis; USA, United States of America.

a Association—Evidence of/or association (univariate/multivariable analysis) with increased severity of influenza disease in those with vs without tuberculosis; or prevalence of co‐infection in those with severe influenza disease.

b Score out of a possible score of 9.

**Table 2:** Summary of studies reporting on the effect of influenza in individuals with tuberculosis (TB)

Study	Country	Year	Descriptive/ analytical	Objective/hypothesis	Setting	Number studied	Influenza type	Laboratory‐confirmed influenza	TB diagnosis	Findings	Association^a^	Newcastle‐Ottawa Score^b^
Dijkman (1967)^26^	Netherlands	1957‐1963	Descriptive	Investigated association between acute respiratory infection (influenza) and unfavourable course of primary pulmonary and hilar tuberculosis among children‐	Tuberculosis sanatorium	36 subjects	Seasonal, influenza A & B	Serology, HI antibodies against influenza A. ≥fourfold increase in antibody titres	Clinical& radiological examination AFB towards end of hospitalization	20% (5/20) of paediatric patients with influenza developed segmental pulmonary lesions. Not evaluated statistically	NA	NA
Sellers (1959)^36^	USA	1957	Descriptive	Assess effect of superimposed viral infection on existing TB	TB sanatorium	31 subjects	Pandemic, influenza A, 1957	HI antibodies to PR8 type A & FMI type A	*Tuberculin* conversion, X‐ray changes ± *M tuberculosis* culture	2/31 TB cases with influenza had signs of worsening on X‐ray (increased perihilar nodes, increased infiltration around cavity and increase in cavity)	NA	NA
Walaza (2015)^32^	South Africa	2010‐2011	Analytical	Compare influenza single infection & influenza‐TB co‐infection to TB single infection	Hospital admission	2959 subjects	Seasonal	RT‐PCR	Microscopy, *M tuberculosis* culture/XPert MTB/RIF	Increased risk of death in cases with co‐infection vs TB only, aRRR 3.1, 95% CI 1.1‐10.1. Increased risk of death in co‐infected vs TB only with symptoms ≥7 d, aRRR 5.5, 95%CI 1.2‐25.3 Adjusted for age, site, HIV status, duration of symptoms, TB treatment, antibiotic therapy, ICU admission, duration of hospitalization HIV infection in TB only vs co‐infected aRRR 1.6 (0.5‐4.9)	Yes	8
Espersen (1954)^28^	Denmark	1952	Analytical	Describe the epidemic of influenza B in a TB sanatorium	Tuberculosis sanatorium	295 subjects	Seasonal, influenza B	HI	Clinical, TB smear ± culture	Radiological changes or sputum conversion in 7/53 (13%) co‐infected vs 3/142 (2%) TB only (*P* = .005)	Yes	6
Dube (2016)^33^	South Africa	2011‐2012	Analytical	Compare prevalence of influenza in children with definite TB to unlikely TB	Hospital admission	214 suspected TB (34 definite TB, 94 Unlikely TB, 86 unconfirmed TB)	Seasonal	Multiplex PCR	*M tuberculosis* culture/XPert MTB/RIF	Influenza C 18% (6/34) vs 4% (4/94), *P* = .04	Yes	6
De Paus (2013)^31^	Indonesia	2001‐2004	Analytical	Did newly diagnosed TB patients have a recent influenza virus infection? Hypothesis—Influenza virus enhanced the susceptibility to develop active TB/ reactivated latent TB	Cases from tuberculosis clinic and community controls	111 TB cases; 111 community controls, matched for age, sex and socio‐economic status	Seasonal (AH3N2/AH1N1)	Serology HI‐IG and IGM antibodies against influenza A HI titre ≥10	WHO case definition (clinical + CXR changes positive microscopy and culture for Mtb)	Prevalence of influenza antibodies among TB cases vs controls was 46% vs 41% (*P* = .5) for A/H1N1pdm09 and 82% vs 82% (*P* = 1.0) for A/H3N2	No	8
Roth (2013)^30^	Thailand	2003‐2011	Analytical	Compare characteristics of TB/influenza to influenza and TB only	Hospital admissions	7180 subjects	Seasonal and pandemic	RT‐PCR/serology	≥1 sputum AFB or culture positive	Death in 0/23 cases with co‐infection vs 30/646 (4.6%) in cases with TB only, *P* = .6	No	6

Abbreviations: aRRR, adjusted relative risk ratio; CI, confidence interval; CPR, case‐population ratio; NA, not applicable; TB, tuberculosis; USA, United States of America.

a Association—Evidence of/or association (univariate/multivariable analysis) with increased severity of tuberculous disease in those with vs without influenza; or increased frequency of co‐infection vs single infection in those with severe tuberculosis disease.

b Score out of a possible score of 9.

### 3.2 The effect of PTB in patients with influenza

Of the seven studies that reported on PTB in individuals with influenza, six were from HBCs (Table 1)^24^, ^27^, ^29^, ^30^, ^34^, ^35^ and four were analytical studies.^24^, ^27^, ^29^, ^30^

### 3.3 Descriptive studies

Three descriptive studies from HBC using data from the 2009 influenza pandemic reported the prevalence of PTB in individuals with influenza. Two of these studies reported a high frequency of tuberculosis (9% and 10%) in cases hospitalized with influenza and among influenza deaths relative to expected community prevalence. However, no inferences could be made on the significance of the association as there were no comparison groups or data were not evaluated statistically.^34^, ^35^ In a report of individuals that died with influenza A(H1N1)pdm09 virus infection in South Africa, the underlying PTB was identified in seven (10%) of the 72 deaths, which was higher than the 1% general population prevalence in 2006.^34^ Similarly, in a hospital‐based case series of patients positive for influenza A(H1N1)pdm09 virus in India, 9% of influenza cases had PTB compared with 0.4% tuberculosis prevalence in general population (*P* < .001).^35^ In a case series of patients infected with influenza A(H1N1)pdm09 virus in South Korea <1% (7/12 196) had newly diagnosed PTB and there were no deaths among the co‐infected individuals (0/7).^25^

### 3.4 Analytical studies

Three of the four analytical studies were from HBC, including one of high quality that reported no association with severe disease among patients with influenza‐PTB co‐infection compared to patients with influenza only.^24^, ^29^, ^30^ One analytical study reported PTB as a risk factor for influenza‐associated severe acute respiratory illness (SARI) hospitalization.^27^ In this case‐population study from South Africa, tuberculosis was twice as prevalent among hospitalized influenza‐associated SARI cases compared with the general South African population (case‐population ratio [CPR] 1.85, 95%CI 1.68‐2.02).^27^ A case‐control study from Kenya reported that 6% of hospitalized cases with influenza‐associated SARI had PTB compared with <1% of neighbourhood‐matched controls (unadjusted OR 12.0, 95% CI 1.3‐107.37); however, the underlying PTB was not associated with influenza hospitalization on multivariable analysis.^29^ Less than 1% (23/7180) of patients hospitalized for acute respiratory illness and enrolled in a study from Thailand were co‐infected with influenza viruses and tuberculosis. There were no deaths among the 23 cases with influenza‐TB co‐infection, whereas 17 (2.8%) deaths occurred among cases in whom only influenza was identified, *P* = .1.^30^ In a review of 19 cases with laboratory‐confirmed influenza A(H1N1)pdm09 virus infection with respiratory failure admitted to an intensive care facility in South Africa, PTB was present in 4/13 (30%) who died vs 0/6 (0%) who survived *P* = .5.^24^

### 3.5 The effect of influenza in patients with PTB

Of the seven papers that reported data on influenza in patients with PTB (Table 2),^26^, ^28^, ^30^, ^31^, ^32^, ^33^, ^36^ four were from tuberculosis HBCs and the other three were from Europe in a period with high tuberculosis prevalence.^30^, ^31^, ^32^, ^33^ Four of these papers were reported by the authors as analytical studies,^30^, ^31^, ^32^, ^36^ and a fifth^28^ had data suitable for authors of this manuscript to review analytically.

### 3.6 Descriptive studies

Two descriptive studies reported on influenza in cases with tuberculosis housed at a sanatorium. Of these, one study described the effect of seasonal influenza on tuberculosis disease progression and complications.^26^ This study, from the Netherlands in 1967, among children institutionalized with primary tuberculosis of the lungs and hilar lymphadenopathy reported a high frequency of developing secondary segmental pulmonary lesions, suggesting progression of PTB following serologically diagnosed influenza virus infection (defined as greater than fourfold rise in anti‐influenza virus antibody titres).^26^ The other study in a tuberculosis sanatorium in the United States described the effect of superimposed viral infection on existing tuberculosis following an outbreak of the 1957 influenza A pandemic virus, in which two of 31 TB paediatric cases with influenza infection had evidence of worsening of tuberculosis on chest radiography (Table 2).^36^

### 3.7 Analytical studies

In an observational study from South Africa, hospitalized cases with influenza‐PTB co‐infection compared to cases with tuberculosis only had increased risk of death (adjusted relative risk ratio [aRRR 3.1, 95% CI 1.1‐10.1]). This association was, however, only observed in patients with symptoms ≥7 days (aRRR 5.5, 95% CI 1.2‐25.30) and not in cases with symptoms <7 days (aRRR 0.9, 95% CI 0.1‐8.6).^32^ In a case series during an influenza B epidemic in a Danish tuberculosis sanatorium, 13% (7/53) of individuals co‐infected with influenza viruses compared to 2% (3/142) of individuals with tuberculosis only developed tuberculosis complications which included radiological changes or sputum conversion back to being positive (*P* = .005).^28^ Among children admitted with suspected tuberculosis in a study from South Africa, a higher prevalence of influenza C was detected in children with laboratory‐confirmed PTB compared with unlikely tuberculosis (18% [6/34] vs 4% [4/94], *P* = .04).^33^ A case‐control study from Indonesia investigating the putative association between tuberculosis and influenza virus infection reported no association between the development of clinically active PTB, either through reactivation of latent tuberculosis or directly after exposure to *M tuberculosis*, and influenza virus infection as measured by unpaired serology in cases with newly diagnosed tuberculosis and community controls. The proportion of individuals with influenza virus antibody titres ≥10 against influenza A(H3N2) and A(H1N1) viruses in patients with tuberculosis were similar to matched community controls; however, the antibody titre levels for influenza A(H3N2) virus at time of tuberculosis diagnosis were significantly higher (1.7 times higher, *P* = .002) in cases with PTB compared to controls. In addition, the difference in titres between cases with advanced PTB on chest X‐ray and their controls was significantly higher than in cases with mild to moderate tuberculosis and their controls.^31^ Among 23 patients with concurrent PTB and influenza infection from Thailand, none died, compared with 30 (4.7%) deaths among the individuals with only tuberculosis; however, this was not statistically significant (*P* = .62).^30^

### 3.8 Summary of quality of human studies

Of the eight analytical studies, three were high‐quality studies as assessed by the Newcastle‐Ottawa score, of which one showed an association between influenza‐PTB co‐infection and increased mortality compared with tuberculosis only^32^ and two showed no association between co‐infection and severe influenza disease^29^ or correlation between influenza infection and tuberculosis.^31^ Over a third of the studies about PTB and influenza virus co‐infection were descriptive case series that included univariate analysis, and the causal relationship could not be demonstrated. Some of the studies used clinical criteria for PTB cases; however, the specifics of the criteria used were not always fully described. Among the studies that included laboratory‐confirmed PTB, screening for tuberculosis was not done systematically.

### 3.9 Summary of findings from experimental animal models

In murine models, five studies suggested that influenza and tuberculosis co‐infection affected tuberculosis and influenza disease presentation or outcome,^20^ and one study showed no effect (Table 3).^37^ Five of the murine studies reported on the effect of influenza on tuberculosis, and one study reported on the effect of influenza on tuberculosis and the effect of tuberculosis on influenza.

**Table 3:** Summary of experimental animal studies exploring the interaction between influenza and tuberculosis

Reference	Objective	Experiment	Influenza strain/TB	Period/ observation period	Findings
Volkert (1947)^20^	Does viral infection of the lungs superimposed on tubercle bacilli infection alter cause and outcome of infection due to bacterium	Model: Mice 1. Experiment group 1 Experimental group Simultaneous TB bacilli given intraperitoneally and influenza A virus (PR8) intra nasally Control group 1: Inoculated with TB only Outcomes: Number of gross tuberculosis pulmonary lesions measured at 3 wk Experiment group 2: Inoculation with TB bacilli and influenza 3 wk later Outcomes: Number of gross TB pulmonary lesions measured 6 wk after TB infection (3 wk after influenza challenge)	Influenza A PR8/ culture of tubercle bacilli	3 and 6 wk	Experimental group 1 Increased number and extent of pulmonary lesions compared to control group Experimental group 2: Increased number and extent of pulmonary lesions compared to control group
Florido (2013)^38^	Assessed impact of influenza A virus and mycobacterial respiratory co‐infection on development of CD8 T‐cell responses to each pathogen	Experiment 1 Experimental group: *Mycobacterium bovis* bacille Calmette‐Guerin (BCG) and influenza A/PR8 at D1 Control groups: BCG only at D1 Outcomes: Number of BCG and influenza‐specific CD4 and CD8 T cells, number of mycobacteria, viral titres, and number of leucocytes at D7, D14 and D21 post‐infection Experiment 2: Experimental group: BCG at D1, and influenza infection at 7 wk Control group: BCG at D1, TB treatment at week 3 (for 4 wk) and influenza infection at 7 wk Outcomes: BCG‐specific CD8 T‐cell response D21 post‐influenza	Influenza A/ PR8; BCG	Experiment 1:7, 14, 21 d post‐infection; Experiment 2 21 d post‐influenza challenge (challenge at 7 wk)	Experiment 1 Experimental (co‐infected) group—reduced frequency and magnitude of BCG‐specific CD8 T cells in the lungs and reduced magnitude of BCG‐specific CD4 and CD8 T cell IFNγ—secreting responses; no difference in influenza‐specific CD8 T cells Co‐infected group had increased number of viable BCG ova Co‐infected group had more extensive/persistent leucocyte accumulation Experiment 2‐ Experimental group had reduced BCG‐ specific CD8 T‐cell response
Redford (2014)^39^	1. Effect of prior IAV on susceptibility to tuberculosis 2. Effect of IAV/*M tuberculosis* co‐infection on control of TB	Model: mice 1. Experimental group: Intranasal IAV on D1, aerosolized MTB on D28 Control group: Intranasal placebo (phosphate‐buffered saline [PBS]) on D1, MTB on D28 Outcomes: lung inflammation, survival, number of viable bacteria in lung tissue 2. Experimental group 2: Aerosolized MTB D1, intranasal IAV (subtype (Cal/09) on D1 and IAV (subtype X3) on D14 Control group: Aerosolized MTB D1, placebo (PBS) on D1 and D14 Outcomes: Number of viable MTB measured on D27	Influenza A Virus/ *M tuberculosis*	Model 1:120 d	Model 1 Experimental group 1 had significant increase in inflammation, decreased survival, higher number viable MTB in lung compared to the control group
				Model 2:27 d	Model 2 Experimental group had significantly increased mycobacterial load compared to control group
Bernard, (1962)^37^	Assessed the effect of influenza infection on TB‐ infected mice. Measured TB bacilli per nodule in the sacrificed mice and time from infection to death for the mice that were not sacrificed	Model: Mice Experimental group Groups 1, 2, 3, 4 and 5 (10 mice in each) TB challenge at week 1, 2, 3, 4 and 5, respectively. Influenza challenge at week 6. 50% of mice sacrificed 15 d post‐influenza challenge Group 6 (20 mice)‐ influenza only challenge at week 6 for all; Not sacrificed Control groups Groups 1, 2, 3, 4 and 5 (10 mice in each) TB challenge at week 1, 2, 3, 4 and 5, respectively. No influenza challenge Outcomes: Number of TB bacilli per nodule in the sacrificed mice Time from TB infection to death for the mice that were not sacrificed	H 37 RV strain of TB	173 d, 50% of mice in experimental groups 1‐5 sacrificed 15 d post‐influenza challenge	Experimental groups had 50%‐75% lower survival time and had increased number of bacilli per nodules. Effect of influenza infection on TB severity increased with increasing duration of TB infection before influenza challenge Among non‐sacrificed mice, death in 25/25 (100%) co‐infected vs 1/20 (5%) infected with influenza only
Massanari (40)	Examined tuberculin hypersensitivity during superimposed acute influenza infection	Model: Mice Experimental groups 1: Influenza virus, intranasal (i.n.) 4‐6 wk after *M tuberculosis* infection Experimental group 2 Formalin‐inactivated influenza virus (i.n.) or intravenous (i.v.) live influenza virus 4‐6 wk after *M tuberculosis* infection Control group Inoculated with PBS, 4‐6 wk after *M tuberculosis* infection Outcomes: Tuberculin hypersensitivity (measured as footpad swelling) 6 d after influenza, PBS challenge Number of circulating lymphocytes post‐influenza challenge	*M tuberculosis* and influenza A/PR8	4‐6 wk after TB infection	Experimental group 1 Suppressed tuberculin hypersensitivity from D3 to D16 post–intranasal influenza infection. Suppression of immune response preceded presentation of clinical signs of influenza Reduction in lymphocytes on D 2, 5, 7 post‐influenza challenge compared to values before influenza infection Experimental group 2 No immunosuppression in experimental group 2
Co (2006)^41^	Tested how BCG‐specific and influenza‐specific CD4 T cells distribute between the two inflammatory sites (lungs and liver), how these two T cells would interfere with each other and how these interactions affect granuloma formation, dissemination, and control of BCG	Model: Mice Experimental groups: Mice chronically infected with BCG and intranasal hen egg lysozyme (HEL‐flu) influenza challenge 5 wk post‐BCG Mice chronically infected with BCG and wild type (wt) influenza challenge 5 wk post‐BCG Control group: Mice infected with BCG alone Outcome: Number of AFB per lesion, number of granuloma lesions and dissemination of mycobacteria from granuloma	HEL‐flu or wt. influenza virus/ BCG	5 wk 6 d	Experimental groups Increase in numbers of granulomas/field (granuloma burden) in experimental groups compared to controls Slight increase in number of AFB per lesion compared to the control group No dissemination of mycobacteria from granuloma Interpretation: Co‐infection with influenza had little effect on mycobacterial load mycobacteria did not disseminate in either group

Abbreviations: BCG, Calmette‐Guerin; HEL, hen egg lysozyme; IAV, influenza A virus; IFN, interferon; *M tuberculosis*, *Mycobacterium tuberculosis*; TB, tuberculosis.

### 3.10 Effect of influenza on tuberculosis

Volkert et al^20^ showed that the course of experimental infection with tubercle bacillus in mice was worsened by simultaneous influenza infection (influenza A virus and tubercle bacilli challenge at week 0) and influenza infection superimposed on tuberculosis infection (influenza challenge 3 weeks after TB challenge). Co‐infection resulted in more extensive and rapid development of PTB lesions in mice than infection with tubercle bacillus only. Florido et al^38^ reported that pulmonary bacille Calmette‐Guerin (BCG)‐specific CD8 T‐cell responses were impaired in co‐infected mice. Concurrent infection of mice with influenza virus and BCG (challenge on day 0) and sequential infection of mice with TB and influenza virus (TB infection on day 0 and influenza virus 7 weeks later) compared to infection with BCG only resulted in reduction in BCG‐specific CD4 and CD8 T‐cell responses, increased pulmonary disease and a delay in mycobacterium clearance from the lungs of infected mice. For sequential infection with influenza, the reduction in BCG‐specific CD8 T‐cell response was only evident in mice with untreated TB compared with mice that had cleared TB. Concurrent infection with influenza virus and tuberculosis reduced generation of protective T‐cell responses against intracellular mycobacteria but did not affect control of pulmonary influenza viral loads (no difference between co‐infected mice compared with the influenza only group).^38^

Redford et al^39^ demonstrated that influenza A virus infection of mice 28 days before or during (on day 1 or day 14) *M tuberculosis* infection enhanced susceptibility to tuberculosis and impaired mycobacterium control and decreased host survival. Bernard et al^37^ showed that in *M tuberculosis*‐infected mice, influenza virus challenge 1‐5 weeks after *M tuberculosis* infection, compared with *M tuberculosis*‐only infected mice, resulted in 50%‐75% shorter survival time and a higher case‐fatality rate. In addition, the effect of influenza virus on tuberculosis severity, measured by amount of tissue damage, increased with increasing time of tuberculosis infection prior to the influenza virus challenge. Five per cent of the mice infected with influenza alone died compared to 100% of the mice infected with influenza and tuberculosis. This was corroborated in a study by Bernard et al^37^ in which 5% of the mice infected with influenza alone died compared to 100% of the mice infected with influenza and tuberculosis (Table 3). 

Massanari (40) reported that tuberculin hypersensitivity in mice was temporarily suppressed following an intranasal influenza virus challenge; however, a normal response resumed after resolution of influenza virus infection. Tuberculin hypersensitivity, tested 4‐6 weeks after tuberculosis infection, was temporarily suppressed from day 3 to day 16 post an intranasal influenza virus challenge.^40^ In contrast, Co et al^41^ showed that influenza viruses had little effect on mycobacterial load and did not affect dissemination of tuberculosis in a mouse model. They showed that T cells responding to an acute influenza virus infection can modulate host responses to an ongoing BCG infection. Though not statistically significant, acute infection with influenza in mice with chronic *Mycobacterium bovis* BCG infection moderately increased the acid‐fast bacilli load in the liver.^41^

## 4 DISCUSSION

Our systematic review suggests that analytical studies exploring the interaction between laboratory‐confirmed influenza virus infection and clinically diagnosed or laboratory‐confirmed PTB are severely limited. Experimental animal studies suggest an association, specifically that influenza‐tuberculosis co‐infection in mice results in more severe disease than influenza only or tuberculosis only disease. Observational studies among humans showed mixed results. Fifty per cent (4/8) of the analytical studies, one of which was of high quality, showed an association between co‐infection and severe influenza‐ or tuberculosis‐associated disease or increased prevalence of influenza co‐infection in individuals hospitalized for tuberculosis.^27^, ^28^, ^32^, ^33^ The other half (4/8) of the analytical studies, two of which were of high quality, showed no association between co‐infection and progression of tuberculosis or influenza disease or severe outcomes, that is they did not show that influenza affected PTB presentation and outcomes, or that PTB affected influenza presentation and outcomes. Three of the descriptive studies, although not assessed for statistical significance, reported either a high prevalence of co‐infection in cases with severe influenza disease^34^, ^35^ or increased severe disease or progression of disease in co‐infected individuals.^26^

Of the five studies reporting on pandemic influenza only, two descriptive studies from HBCs reported a high prevalence of tuberculosis in cases with severe influenza‐associated disease.^34^, ^35^ These studies presented limited univariate analyses. Pandemic influenza may behave differently to seasonal influenza because of lack of pre‐existing immunity, and the likely interaction between influenza and tuberculosis might be immunologically mediated. High levels of cytokines produced as part of the inflammatory response to infection with a pandemic virus have been reported to result in severe influenza‐associated lung damage.^42^ Some studies have demonstrated a higher mortality due to 2009 pandemic influenza as compared to seasonal influenza.^43^, ^44^, ^45^ High‐quality epidemiological studies are required to assess whether the severe disease and outcomes associated with influenza‐PTB co‐infection are driven by pandemic phenomena as this may have implications for recommendations and prevention strategies. However, even if the association between influenza and PTB is less marked during seasonal influenza epidemics, targeting individuals with active PTB for influenza vaccination and antiviral treatment in HBCs could still potentially prevent significant morbidity and mortality and might also prevent further spread of tuberculosis during the intensive phase if influenza increases coughing. In this review, all the analytical studies were conducted in high burden countries. It is important to understand the background prevalence of tuberculosis where studies are conducted for better interpretation of the results. In countries with low tuberculosis burden, it is possible for studies not to identify increased prevalence of co‐infection or detect an association between co‐infection and severe outcomes, purely because of low numbers due to low tuberculosis prevalence in the community. In some of the analytical studies included in our review, lack of association may be due to the inclusion criteria, for example only including lower respiratory tract infection (LRTI) cases with acute presentation and not systematically testing for PTB in patients with severe respiratory illness. Depending on the magnitude of tuberculosis burden, results may have different implications for prioritization in different settings.

Since the 1950s, authors have recommended influenza vaccination among patients with tuberculosis during influenza epidemics.^28^ Influenza vaccination is the most effective way to prevent influenza‐associated disease. Influenza vaccine has been shown to generate antibody response in patients with tuberculosis that is similar to those without tuberculosis, although these studies were conducted in the 1950s and 1960s and did not include HIV‐infected individuals.^36^ Antiviral treatment for influenza improves outcomes for patients with severe influenza‐associated disease.^46^ However, both vaccines and antiviral treatment have cost implications and are not easily accessible in low‐ to middle‐income countries where the burden of tuberculosis and influenza are high.^47^ Identifying PTB patients as a risk group for severe influenza‐associated disease may assist policymakers in making decisions about prioritizing this group of patients for influenza vaccination and treatment with influenza antiviral treatment. More high‐quality epidemiological data from high tuberculosis burden settings are needed to address this question. In addition, more studies are needed to determine whether seasonal or pandemic vaccines or influenza antivirals should be prioritized for PTB patients and whether patients hospitalized with influenza‐associated illness should be investigated for PTB.

Although some descriptive and analytical studies inferred worsening of PTB in co‐infected individuals,^26^, ^36^, ^48^ besides the methodological limitations of the studies, changes reported could have simply reflected a superimposed viral or bacterial infection in cases with the underlying PTB rather than worsening of tuberculosis. In addition, there was no comparison of radiological findings in patients with and without co‐infection to assess whether changes in the lungs were a factor in the presentation or outcomes of influenza‐associated disease. The one study which showed an increase in pulmonary lesions did not present results on whether the radiological changes correlated with deterioration in clinical presentation.

One of the studies suggested that compared to individuals infected only with tuberculosis, individuals with influenza‐PTB co‐infection had increased risk of death, and this association was not observed in patients with a more acute presentation. If cases with more chronic PTB are more at risk of severe influenza disease, this might explain the lack of association in some of the studies which only included LRTI patients with an acute presentation.^29^, ^30^ If the association with severe disease and poor outcomes is more prevalent in patients with a more chronic presentation, this may further assist in making decisions about which tuberculosis cases to prioritize for interventions, especially in countries where the tuberculosis burden is high and resources are limited.

The mechanism by which influenza‐PTB co‐infection leads to severe influenza‐associated disease may be secondary to the underlying lung damage caused by PTB. It is possible that those who had severe outcomes from co‐infection already had the underlying lung damage from PTB leading to reduced lung capacity to deal with a viral infection such as influenza. Seki et al^49^ suggested that the underlying chronic lung diseases such as tuberculosis may be an important factor in the increase in frequency of secondary bacterial pneumonia in persons with influenza, which in turn can lead to increased frequency of complications.

Some of the studies reported on tuberculosis in patients from sanatorium.^26^, ^28^, ^36^ It is possible that the high prevalence of influenza reported in these studies is due to increased risk of influenza transmission resulting in high transmission rates in these closed settings. In addition, the influenza transmission may not reflect community‐acquired influenza and results from these studies cannot be generalizable to other settings. Due to a possibility of increased risk of high concentration of persons with co‐morbidities resulting in poor outcomes, closed settings should be prioritized for influenza vaccination.

There were a number of limitations to this systematic review. Broad search terms were used to increase sensitivity to identify relevant articles, although this may have somewhat reduced search specificity. Over a third of the observational studies were descriptive, and due to the nature of these studies, an association could not be evaluated. The type of tuberculosis included differed among the studies, with some studies reporting newly diagnosed tuberculosis, some reporting on cases in a tuberculosis sanatorium for a number of months and some included cases who had completed tuberculosis treatment, thus making data less comparable. There were differences in the population tuberculosis incidences where studies were conducted which could affect the power to detect an association. However, the majority of studies were from tuberculosis high burden countries or were conducted during the period when tuberculosis burden was high. We included animal studies although these may not be generalizable to humans.

Many studies did not adequately assess the underlying conditions such as HIV and malnutrition. HIV infection is a risk factor for severe influenza disease as well as for PTB, and it is an important contributor to the overall burden of severe influenza in high HIV‐prevalence settings.^50^, ^51^ However, only a few papers reported data on HIV infection.^27^, ^29^, ^30^, ^32^ In one study, patients with co‐infection of HIV and PTB were at high risk of being hospitalized with influenza; however, the number of co‐infected individuals was low and the association was not statistically significant.^29^ If the association with severe disease is higher in patients with the underlying HIV infection, it may be difficult to differentiate the role played by the individual infection. Other conditions such as malnutrition, which like tuberculosis are prevalent in HBC, were not evaluated in included articles and may be confounders in the association between influenza and PTB. The numbers of participants in most studies were small, and this could have limited the ability to detect significant associations. Other important areas that were not addressed by the studies reviewed include whether influenza infection caused reactivation of latent tuberculosis or whether the acute viral infection precipitated a visit to the doctor in patients who already had tuberculosis disease. Studies in Chinese were not included in the review, and therefore, our review may not reflect the full body of literature on this topic.

## 5 CONCLUSION

Although the majority of experimental animal studies suggested increased severity of disease with co‐infection of influenza and PTB, only half of the analytical studies on influenza and PTB in humans found the same. Descriptive studies, although they could not evaluate an association, reported an increased prevalence of co‐infection among cases with severe influenza or PTB disease. Data are limited from large epidemiological studies, studies with laboratory‐confirmed influenza and PTB, studies from high tuberculosis burden settings and studies that include data on HIV. In order to study the association between influenza and PTB and make inferences about causal associations, more epidemiological studies with systematic testing for influenza and tuberculosis are needed.

## CONFLICT OF INTEREST

No authors have any competing interests.

## AUTHOR CONTRIBUTIONS

SW and ALC conceived and designed the experiments; SW, ST, MM, AN, CC, JM and ALC performed the experiments and analysed the data; SW, ST, MM, AN, CC, SAM, JM and ALC wrote and reviewed the paper.

## DISCLAIMER

The findings and conclusions in this report are those of the authors and do not necessarily represent the official position of the US Centers for Disease Control and Prevention.
