# Supplementary material for: Influenza and tuberculosis co‐infection: A systematic review
Source: Influenza Other Respir Viruses. 2019 Sep 30;14(1):77–91. doi: 10.1111/irv.12670 (PMC6928059; doi:10.1111/irv.12670)
Supplement: Supplementary file 1 [file IRV-14-77-s001.doc]

**Influenza and Tuberculosis**

Time period covered: January 01, 1900- December 31, 2018

**Databases used:**

Medline

Embase

PsycInfo

CINAHL

Scopus (previously, Web of Science)

Cochrane

CAB Abstracts and Global Health

**MEDLINE**

**I. Influenza**

exp Influenza, Human/ or exp influenza a virus/ or exp influenzavirus b/ or influenza or flu

**II. TB**

exp Tuberculosis/ or tuberculosis

**EMBASE**

**I. Influenza**

exp Influenza virus/ or exp Influenza virus A/ or exp Influenza virus B/ or exp INFLUENZA B/ or (influenza or flu).ti,ab

**II. TB**

exp Tuberculosis/ or tuberculosis

**PSYCINFO**

**I. Influenza**

exp Influenza/ or influenza or flu

**II. TB**

exp Tuberculosis/ or tuberculosis

**CINAHL**

**I. Influenza**

(MH "Influenzavirus A+") OR (MH "Influenzavirus B+") OR (MH "Influenza A Virus+") OR (MH "Influenza B Virus") OR (MH "Influenza, Human+") or influenza or flu

**II. TB**

(MH "Tuberculosis+") or tuberculosis

**SCOPUS**

**I. Influenza**

TITLE-ABS-KEY (influenza or flu)

**II. TB**

TITLE-ABS-KEY (tuberculosis)

**COCHRANE**

**I. Influenza**

#1 MeSH descriptor Influenza, Human explode all trees

#2 MeSH descriptor Influenza A virus explode all trees

#3 MeSH descriptor Influenzavirus B explode all trees

#4 (influenza or flu).ti,ab,kw

#5 (#1 OR #2 OR #3 OR #4)

**II. TB**

MeSH descriptor Tuberculosis explode all trees

or

(tuberculosis).ti,ab,kw

**GLOBAL HEALTH/CAB ABSTRACTS**

**I. Influenza**

(influenza or flu).ab,ti.

**II. TB**

(tuberculosis).ab,ti.
