# Supplementary material for: Influenza and tuberculosis co‐infection: A systematic review
Source: Influenza Other Respir Viruses. 2019 Sep 30;14(1):77–91. doi: 10.1111/irv.12670 (PMC6928059; doi:10.1111/irv.12670)
Supplement: Supplementary file 2 [file IRV-14-77-s002.docx]

**Influenza and tuberculosis co-infection: a systematic review**

Sibongile Walaza, Cheryl Cohen, Stefano Tempia, Jocelyn Moyes , Athermon Nguweneza, Shabir A. Madhi, Meredith McMorrow, Adam L. Cohen

**Corresponding author**:

Dr Sibongile Walaza

Sibongile Walaza, Centre for Respiratory Diseases and Meningitis, National Institute for Communicable Diseases, Private Bag X4, Sandringham, 2131, Gauteng, South Africa. Email: [sibongilew@nicd.ac.za](mailto:sibongilew@nicd.ac.za)

**Review Question**

To perform a systematic review to summarise whether individuals with influenza-tuberculosis coinfection present with severe influenza or pulmonary tuberculosis disease as compared to those with single infection or disease.

**Searches**

Include search terms for influenza (“influenza” or “flu or “influenza virus” or “human influenza” or “influenza vaccine” or influenza vaccination”) and for tuberculosis (“tuberculosis” or “TB”). The Medline, Embase, PsycINFO, CINAHL, Web of Science, Cochrane, CAB Abstracts and Global Health databases will be searched systematically. In addition, bibliographies of papers that are reviewed will be checked for further relevant publications. The search will be restricted to articles published in English, French, Italian, German, Russian, Finish, Japanese or Portuguese. This review will be restricted to published abstracts and articles from January1900 to December 2018 that report data on the association (burden of disease, transmission and severity) between laboratory-confirmed influenza and clinically diagnosed or laboratory-confirmed pulmonary tuberculosis.

**Type of studies to be included**

For human studies, inclusion will be limited to studies in which influenza is laboratory-confirmed and tuberculosis includes pulmonary tuberculosis disease. Animal studies will be included as they may provide useful insights into possible underlying mechanisms of interactions in humans.

Studies that modelled ecological data on the association between influenza and tuberculosis, individual case reports, vaccine studies and influenza antiviral therapy in patients with tuberculosis will be excluded.

**Condition or domain being studies**

Influenza and tuberculosis coinfection and its impact on disease presentation and severity in in children and adults.

**Participants/population**

Participants were restricted to humans with laboratory confirmed-influenza and laboratory -confirmed tuberculosis or clinically diagnosed tuberculosis disease. There were no limitations based on participant age or location of participant recruitment.

**Intervention/s, exposure/s**

Co-infection will be defined as a laboratory- confirmed pulmonary tuberculosis or clinical diagnosis of pulmonary tuberculosis in patients with laboratory-confirmed influenza. Among pulmonary tuberculosis patients (laboratory confirmed or clinically diagnosed) coinfection will be a laboratory-confirmed influenza.

**Comparator/control**

Single infection/disease (i.e. pulmonary tuberculosis or influenza) or community prevalence of tuberculosis.

**Context**

Both influenza virus infections and tuberculosis disease cause substantial annual morbidity and mortality in humans worldwide. Ecological studies and mathematical modelling of epidemiologic data suggest an increase in the frequency of influenza disease or severe influenza-associated disease in individuals with tuberculosis during influenza pandemics, or during seasonal influenza epidemics compared to otherwise healthy individuals. Understanding the interaction between influenza and pulmonary tuberculosis may assist in determining whether individuals with pulmonary tuberculosis should be prioritised for influenza vaccination and treatment with antiviral medications. To the best of our knowledge, no study has previously summarised the available data from epidemiological studies on interaction between influenza and tuberculosis.

**Main outcome**

To identify the frequency of tuberculosis disease in influenza positive patients and frequency of influenza in pulmonary tuberculosis patients. In addition to describe the prevalence of severe outcomes in patients with influenza-tuberculosis coinfection.

**Data extraction**

Literature search results (titles and abstracts) will be screened independently by two authors to identify all citations that possibly meet the inclusion criteria. Full manuscripts of selected citations will be retrieved and assessed independently by one reviewer against the inclusion/exclusion criteria and checked independently by a second reviewer. Disagreements will be resolved through consultation with a third party.

Data extracted from each study will include: year published, study design, type of study (descriptive vs analytical), location of study, period of study, sample size, study setting (hospital/ICU/outpatient), type of influenza testing, tuberculosis testing method (microscopy, culture, polymerase chain reaction (PCR)), results (influenza and tuberculosis), influenza strains, outcome and findings.

**Risk of bias (quality) assessment**

Individual studies will be independently assessed for potential bias or confounding. When studies use either cohort or case-control designs, we will use the Newcastle-Ottawa Scale to rate the quality of the included papers. Assessment will be done by two independent reviewers.

**Strategy of data synthesis**

Summary measures (odds ratios, relative risks), when reported, will abstracted. Data synthesis will consist of reporting the key findings of the different studies. Where possible the studies will be classified according to whether they fall among the 22 high tuberculosis burden countries that account for ~80% of world’s tuberculosis cases.

**Analysis of subgroups or subsets**

None planned

**Contact details for further information**

Dr S Walaza, sibongilew @nicd.ac.za

**Organisational affiliation of review**

Centre for Respiratory Diseases and Meningitis, National Institute for Communicable Diseases of the National Health Laboratory Service, Johannesburg, South Africa

[**www.nicd.ac.za**](http://www.nicd.ac.za)

**Review team members and their organisational affiliations**

Sibongile Walaza^1,2^, Cheryl Cohen^1,2^, Stefano Tempia^3,4;^, Jocelyn Moyes^1,2^ Athermon Nguweneza^1^, Shabir A. Madhi^1,6,7^; Meredith McMorrow^3,4,5^, Adam L. Cohen^3,4,8^

**Affiliations**

1. Centre for Respiratory Diseases and Meningitis, National Institute for Communicable Diseases of the National Health Laboratory Service, Johannesburg, South Africa
2. School of Public Health, Faculty of Health Sciences, University of the Witwatersrand, Johannesburg, South Africa
3. Influenza Division, Centers for Disease Control and Prevention, Atlanta, Georgia, United States of America
4. Influenza Program, Centers for Disease Control and Prevention, Pretoria, South Africa
5. U.S. Public Health Service, Rockville, Maryland, United States of America
6. Medical Research Council, Respiratory and Meningeal Pathogens Research Unit, University of the Witwatersrand, Johannesburg, South Africa
7. Department of Science and Technology/National Research Foundation: Vaccine Preventable Diseases, University of the Witwatersrand, Johannesburg, South Africa
8. Global Immunization Monitoring and Surveillance, Expanded Programme on Immunization Department of Immunization, Vaccines and Biologicals World Health Organization

**Anticipated/Actual start date of the review**:

03 January 2015

**Anticipated completion date:**

March 2019

**Funding Sources/sponsor**

None. Since publicly available data will be used, this research will not receive any specific grant from funding agencies in the public, commercial, or non- profit sectors.

**Conflict of interest**

None Known

**Language**

English

**Country**

South Africa
